# Self-Compliant Bipolar Resistive Switching in SiN-Based Resistive Switching Memory

**DOI:** 10.3390/ma10050459

**Published:** 2017-04-26

**Authors:** Sungjun Kim, Yao-Feng Chang, Min-Hwi Kim, Tae-Hyeon Kim, Yoon Kim, Byung-Gook Park

**Affiliations:** 1 Department of Electrical and Computer Engineering, Inter-University Semiconductor Research Center (ISRC), Seoul National University, Seoul 08826, Korea; thinlizzy@snu.ac.kr (S.K.); minboysky@naver.com (M.-H.K.); taehyun4902@snu.ac.kr (T.-H.K.); 2 Department of Electrical and Computer Engineering, The University of Texas at Austin, Austin, TX 78758, USA; u9120009@gmail.com; 3 Department of Nanoenergy Engineering, Pusan National University, Busan 46241, Korea; yoonkim@pusan.ac.kr

**Keywords:** memory, resistive switching, self-compliance, silicon nitride

## Abstract

Here, we present evidence of self-compliant and self-rectifying bipolar resistive switching behavior in Ni/SiN*_x_*/n^+^ Si and Ni/SiN*_x_*/n^++^ Si resistive-switching random access memory devices. The Ni/SiN*_x_*/n^++^ Si device’s Si bottom electrode had a higher dopant concentration (As ion > 10^19^ cm^−3^) than the Ni/SiN*_x_*/n^+^ Si device; both unipolar and bipolar resistive switching behaviors were observed for the higher dopant concentration device owing to a large current overshoot. Conversely, for the device with the lower dopant concentration (As ion < 10^18^ cm^−3^), self-rectification and self-compliance were achieved owing to the series resistance of the Si bottom electrode.

## 1. Introduction

It is projected that, in the near future, NAND flash will be faced with scaling issues caused by cell-to-cell interference, hot carrier disturbance, and word-lines resistance [1]. Because overcoming these inherent physical limitations would cause the complexity of the fabrication process to increase, memory devices based on the new concept of resistive switching have been proposed [2,3,4,5,6,7,8,9,10]. Resistive-switching random access memory (RRAM), with its various redox-related chemical effects, is one of the most promising devices for future non-volatile memory applications because of its superior memory performance and scalability down to a few nanometers [11,12,13,14,15]. The cross-point array structure is the most effective structure for high-density memory applications [16,17,18]. Each memory cell is placed at the intersection points of the bit-lines and word-lines, which are perpendicular to each other. Unfortunately, the sneak current paths from the cells in a low-resistance state (LRS) can limit the array size [16,17,18]. Therefore, additional nonlinear elements, so-called selection devices, are required to suppress the leakage paths in a cross-point array. Many approaches for combining memory and nonlinear selection devices have been proposed [19,20,21,22]. Each approach has some disadvantages: an Si PN diode cannot provide a bidirectional selector function. Insulator-metal-transition (IMT) switching using VO*_x_* is unsuitable in the sense that the selector performance is not maintained at high temperatures [23]. The switching mechanism in varistor-type and mixed-ionic-electronic-conduction (MIEC) selectors is not clear. When an Si bottom electrode (BE) is used in place of a conventional metal BE, nonlinear current-voltage (I-V) characteristics are achieved without additional selection devices [24,25]. The built-in selector function helps increase the nonlinearity in a cross-point array. RRAM devices with a metal-insulator-semiconductor (MIS) structure display unique resistive switching behaviors depending on the dopant type (i.e., n-type or p-type) and the concentration of impurities in the Si BE. Previously, we investigated SiN-based RRAM devices with diode-like self-rectification behavior in unipolar switching mode [26]. Even though unipolar switching has the advantage that all switching occurs in one direction, it also has the disadvantages of a relatively larger variation in the switching parameters and higher reset current, which are both major hurdles to RRAM commercialization. Having a RRAM device with self-compliance would simplify the driving circuit required to limit the current since the low-resistance state (LRS) can be controlled without external compliance. Moreover, self-compliance could reduce the reset current. In this study, we compared two devices with different doping concentrations in Si BE. The self-compliant and self-rectifying bipolar resistive switching in an Ni/SiN*_x_*/n^+^-Si device is more favorable for the nonlinear low-current operation than Ni/SiN*_x_*/n^++^-Si device for use in high-density memory applications.

## 2. Experimental

First, we used arsenic (As) ions as n-type dopant ions in the BE of the metal-insulator-semiconductor (MIS) structure. A 10-nm-thick SiO_2_ layer was grown by dry oxidation as the screen oxide before the ions were implanted at densities of 8 × 10^14^ cm^−2^ and 2 × 10^13^ cm^−2^ into single-crystal silicon to fabricate the n^++^ Si and n^+^ Si BEs, respectively. The lattice damage was repaired by annealing at 1000 °C to restore the single-crystal structure and activate the As ions. We deposited 5-nm-thick SiN*_x_* films via low-pressure chemical vapor deposition (LPCVD) at 785 °C after removal of the SiO_2_ layer. The process gas was a mixture of SiH_2_Cl_2_ (dichlorosilane) with a mass flow of 30 sccm and NH_3_ (100 sccm). A 100-nm-thick Ni top electrode was deposited using a thermal evaporator. The patterning was performed through a shadow mask with a diameter of 100 μm. The electrical characterization for each device was conducted using the DC voltage sweep mode of a Keithley 4200-SCS semiconductor parameter analyzer. The voltage bias was applied to the Ni top electrode, while the Si BE was grounded. To analyze the SiN*_x_* material deposited in our laboratory, X-ray photoelectron spectroscopy (XPS) analysis was performed using a Thermo VG ESCA Sigma Probe spectrometer operating at 15 kV and 100 W with a monochromatic Al-Kα radiation source. The calibration of the binding energy scale was set by fixing the C 1s at 284.5 eV.

## 3. Results and Discussion

**Figure 1:**
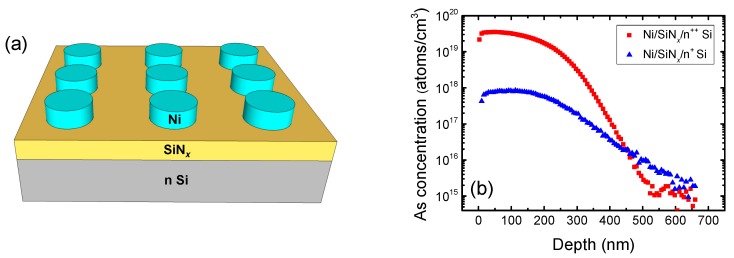

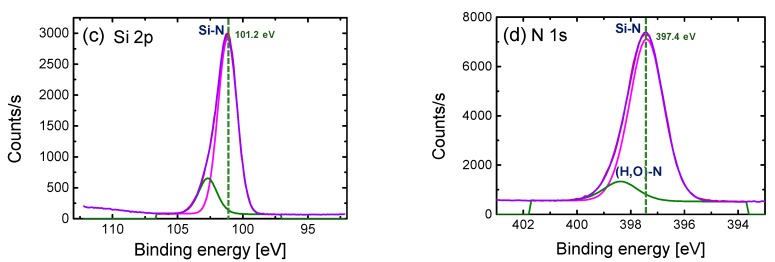
(**a**) Schematic drawing of Ni/SiN*_x_*/n-Si device; (**b**) Secondary ion mass spectrometry depth profile of the Si bottom electrode of the high and low dopant concentration sample; X-ray photoelectron spectroscopy (XPS) spectra of the SiN*_x_* film deposited via low-pressure chemical vapor deposition (LPCVD): (**c**) Si 2p and (**d**) N 1s.

Figure 1a shows a schematic drawing of the Ni/SiN*_x_*/n Si device. Figure 1b shows the depth profiles of the Ni/SiN*_x_*/n^++^ Si and Ni/SiN*_x_*/n^+^ Si devices using secondary ion mass spectrometry. The effective dopant concentration in the BE was above 10^19^ cm^−3^ and below 10^18^ cm^−3^ for the Ni/SiN*_x_*/n^++^ Si and Ni/SiN*_x_*/n^+^ Si device, respectively. Figure 1c,d show the XPS data of the Si 2p and N 1s spectra, respectively, for the SiN*_x_* film deposited by LPCVD. The peak binding energies in the SiN*_x_* film are centered at 101.2 eV and 397.4 eV for the Si 2p and N 1s peak, respectively [27]. The atomic concentration was calculated from the Si 2p and N 1s peaks; the N/Si ratio in the SiN*_x_* was 0.89.

**Figure 2:**
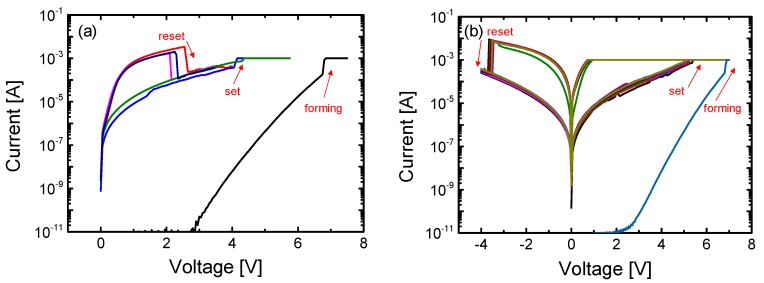
DC current-voltage (I-V) characteristics of the Ni/SiN*_x_*/n^++^ Si resistive-switching random access memory (RRAM) device for (**a**) unipolar switching and (**b**) bipolar switching.

Figure 2a shows the I-V characteristics of the unipolar resistive switching of the Ni/SiN*_x_*/n^++^ Si device. After the electroforming step, reset switching occurs by sweeping from 0 to 4 V, transitioning the device from the LRS to the high resistance state (HRS). The switching voltage of the set process that causes the device transition to the LRS is higher than that of the reset process for same polarity switching. For the formation process, a compliance current (CC) of 1 mA was used to limit the size of conducting paths. Figure 2b shows the I-V characteristics of the bipolar resistive switching of the Ni/SiN*_x_*/n^++^ Si device. After the positive formation process, the reset and set processes occur for a negative and positive voltage sweep, respectively. The absolute set and reset voltages were no different between the unipolar and bipolar switching modes, indicating that Ni/SiN*_x_*/n^++^ Si devices have a non-polar resistive switching property. The conducting paths with LRS (caused by a large overshoot) will be disrupted by Joule heating for both unipolar and bipolar switching.

The intrinsic switching in an SiN-based RRAM device can be induced by the generation and re-passivation of silicon dangling bonds. For the forming and set process, Si–H bonds can be broken by accelerated electrons via thermal effects under high electric fields. Conversely, for the reset process, the resistance increases when hydrogen ions are connected with silicon dangling bonds [28]. 

**Figure 3:**
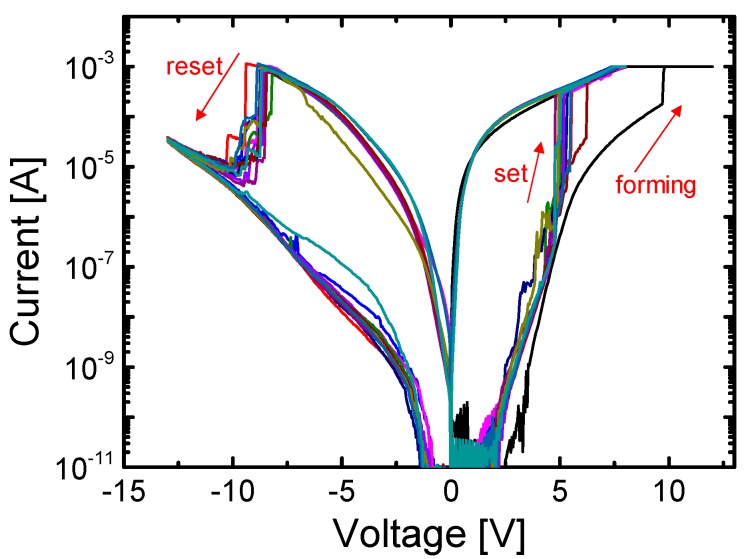
DC I-V bipolar resistive characteristics of the Ni/SiN*_x_*/n^+^ Si RRAM device.

Figure 3 shows the I-V characteristics for bipolar resistive switching of the Ni/SiN*_x_*/n^+^ Si device. A lower operating current than the CC was achieved since the reset switching in the bipolar switching for the Ni/SiN*_x_*/n^+^ Si device is mostly driven by the electric field. Further evidence for the different reset switching mechanisms can also be observed in the form of different reset switching types. Gradual reset switching after abrupt switching, which involves multiple current drops, is observed for the Ni/SiN*_x_*/n^+^ Si device, while the Ni/SiN*_x_*/n^++^ Si device shows an abrupt and one-time current drop.

**Figure 4:**
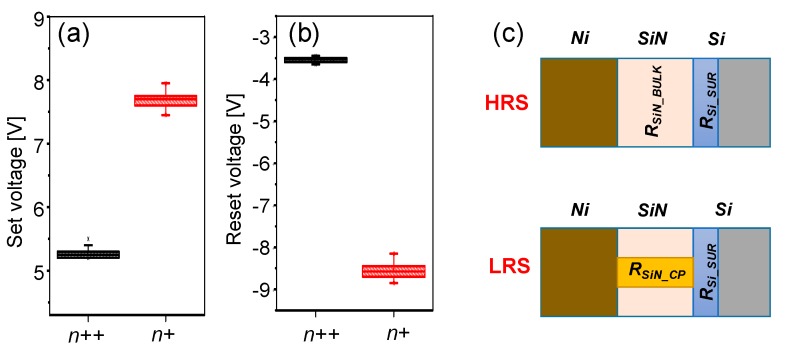
Statistical distribution of (**a**) the set voltage and (**b**) the reset voltage; (**c**) Schematics illustrating the Ni/SiN/Si structure in the high resistance state (HRS) and the low resistance state (LRS).

Figure 4a,b shows the distribution of the switching voltages, including the set and reset voltages, for Ni/SiN*_x_*/n^++^ Si and Ni/SiN*_x_*/n^+^ Si devices. The switching voltage is higher in the Ni/SiN*_x_*/n^+^ Si device than in the Ni/SiN*_x_*/n^++^ Si device. This is attributed to the higher silicon surface resistance (R_Si_SUR_) in the Ni/SiN*_x_*/n^+^ Si device compared with the Ni/SiN*_x_*/n^++^ Si device; namely, R_Si_SUR_ in the Ni/SiN*_x_*/n^+^ Si device acts as a much larger voltage divider than that in the Ni/SiN*_x_*/n^++^ Si device during switching. It should be noted that the reset voltage (V_RESET_) of the Ni/SiN*_x_*/n^+^ Si device is much higher than that of the Ni/SiN*_x_*/n^++^ Si device. The resistance of the SiN film in the LRS is significantly reduced, since the resistance of the conducting paths (R_SiN_CP_) is dominant compared with the bulk resistance of the SiN film (R_SiN_BULK_) in the HRS as shown in Figure 4c. Hence, the active silicon surface resistance (R_Si_SUR_) plays an important role in reset switching compared with set switching. 

**Figure 5:**
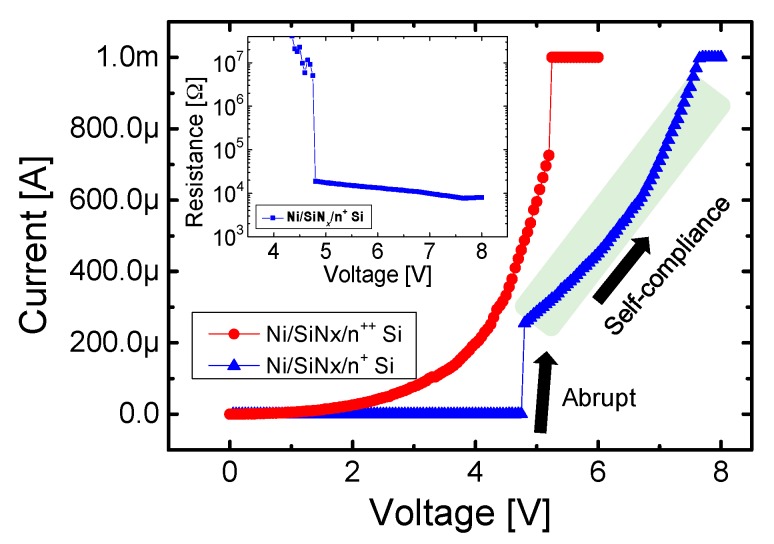
Self-compliance behavior in the LRS for the Ni/SiN*_x_*/n^++^ Si and Ni/SiN*_x_*/n^+^ Si devices.

Figure 5 shows the I-V characteristics of set switching for both devices. Unlike the Ni/SiN*_x_*/n^++^ Si device, the Ni/SiN*_x_*/n^+^ Si device shows self-compliance behavior under 1 mA during the set process. The resistance value slowly decreases with increasing voltage after the abrupt set switching. This autonomous current limitation functionality is caused by the active silicon surface acting as a non-breakable series resistor. 

**Figure 6:**
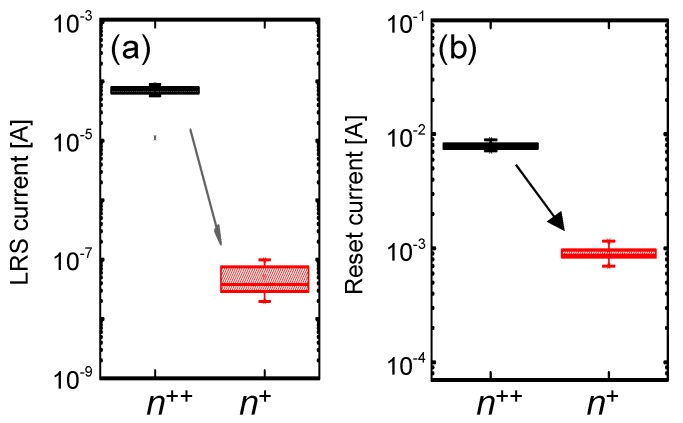
Statistical distribution of (**a**) the LRS current and (**b**) the reset current for Ni/SiN*_x_*/n^+^ Si device and Ni/SiN*_x_*/n^++^ Si devices.

It should be noted that both the LRS current (at a read voltage, V_READ_, of 0.2 V) and the reset current (I_RESET_) of the Ni/SiN*_x_*/n^+^ Si device are lower than those of the Ni/SiN*_x_*/n^++^ Si device, as shown in Figure 6a,b, indicating that the self-compliance can suppress the current overshoot [29,30].

**Figure 7:**
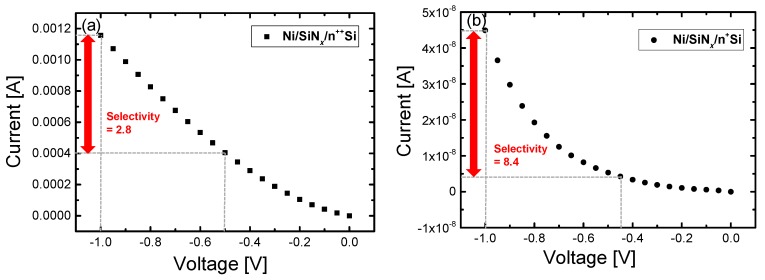
Selectivity in the LRS for (**a**) the Ni/SiN*_x_*/n^++^ Si device and (**b**) the Ni/SiN*_x_*/n^+^ Si device.

To explore the devices’ nonlinear performances, we further investigated the I-V characteristics in the LRS of both devices. The ratio of the current at V_READ_ to that at ½ V_READ_ can be used to evaluate nonlinearity in partial voltage bias schemes such as the ½ V_READ_ scheme; this ratio is called the selectivity from here onwards. Figure 7a,b shows the I-V curves of the Ni/SiN*_x_*/n^++^ Si and Ni/SiN*_x_*/n^+^ Si device in the LRS, respectively, as well as the selectivity of each device. For the Ni/SiN*_x_*/n^++^ Si device, the selectivity was observed to be above 2, indicating that the conducting paths are not perfectly ohmic; in comparison, for metal nitrides such as AlN and NiN, which have strong metallic conducting filaments in the LRS, they show perfectly ohmic conduction [31,32]. This selectivity value of 2 is insufficient to suppress the sneak current paths in a cross-point array. Conversely, a selectivity of over 8 was obtained for the Ni/SiN*_x_*/n^+^ Si device. This is caused by the fact that very narrow conducting paths are more strongly dependent on two electric field-driven tunneling mechanisms, namely trap-assisted tunneling (TAT) and Fowler-Nordheim tunneling (FNT) [33,34,35,36,37].

**Figure 8:**
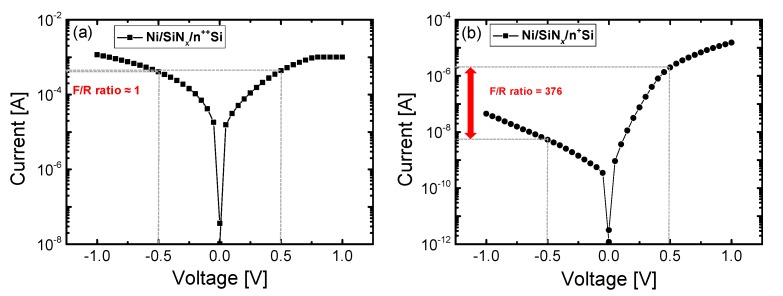
Forward-to-reverse ratio (F/R ratio) in the LRS for (**a**) the Ni/SiN*_x_*/n^++^ Si device and (**b**) the Ni/SiN*_x_*/n^+^ Si device.

Additionally, we calculated the forward-to-reverse ratio (F/R ratio), since the reverse current in the LRS can significantly contribute to reducing the leakage current in a cross-point array [38]. Figure 8a,b shows the I-V curves of the Ni/SiN*_x_*/n^++^ Si and Ni/SiN*_x_*/n^+^ Si devices in the LRS as well as showing the F/R ratios. For the Ni/SiN*_x_*/n^++^ Si device, there are no differences between the forward current at positive voltages and the reverse current at negative voltages, which suggests that the carriers can move freely owing to the low barrier between SiN and Si BE. Conversely, a much higher F/R ratio (>376 at ±0.5 V) can be observed for the Ni/SiN*_x_*/n^+^ Si device. The F/R ratio can be easily affected by the reverse current. 

**Figure 9:**
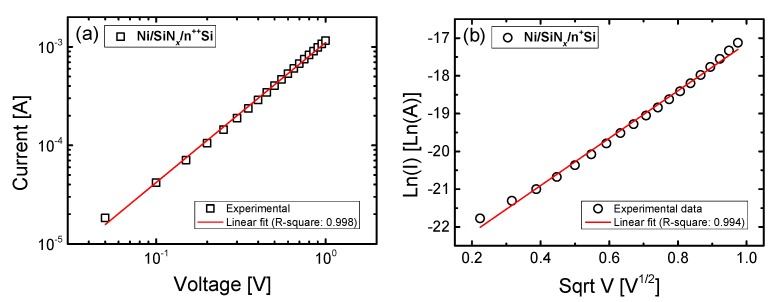
Experimental data and fitting results of the I-V curve in the LRS on (**a**) a log-log scale for Ni/SiN*_x_*/n^++^ Si and (**b**) for ln(I) as a function of sqrt(V) for Ni/SiN*_x_*/n^+^ Si device.

For the Ni/SiN*_x_*/n^++^ Si device, the reverse current region (0 to −1 V) of the LRS is dominated by ohmic conduction (I ∝ V), as can be seen in Figure 9a. Conversely, for the sample with the lower dopant concentration, the reverse current of the LRS is dominated by Schottky emission conducting transport in the reverse current region (0 to −1 V), where ln(I) ∝ V^1/2^ [39].

**Figure 10:**
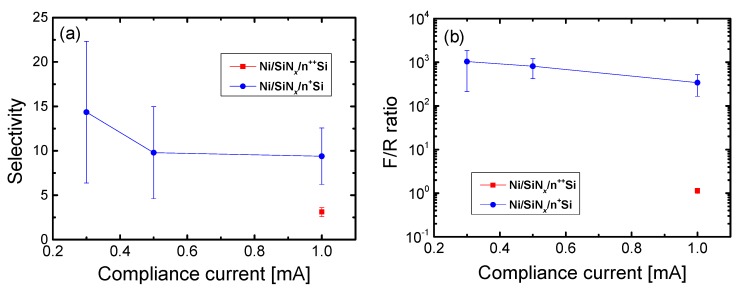
Nonlinearity as a function of the compliance current: (**a**) the selectivity and (**b**) the F/R ratio for Ni/SiN*_x_*/n^+^ Si device and Ni/SiN*_x_*/n^++^ Si devices.

To further enhance the nonlinearity as well as the selectivity and F/R ratio, we can control the conducting paths via the CC. For the Ni/SiN*_x_*/n^+^ Si device, the selectivity can be increased by decreasing the CC, as shown in Figure 10a, which suggests that this intrinsic nonlinear characteristic can be enhanced by narrowing the conducting paths. Figure 10b shows the F/R ratio as a function of the CC. The F/R ratio decreased when the CC was increased. The silicon nitride film can be modulated when the conducting paths are formed during the forming and set processes, causing the Schottky barrier to be lowered. Therefore, a low operating current switching is important in achieving a high nonlinearity of the LRS current. Further studies in the scaling effects on self-rectification and self-compliance are very important for ultra-high-density memory applications. For even smaller device sizes, the rectification behavior would be maintained and self-compliance would be observed at a lower current level owing to lower initial conducting defects in smaller cell sizes.

## 4. Conclusions

In summary, we investigated the self-compliant and self-rectifying bipolar resistive switching characteristics of Ni/SiN*_x_*/n^+^ Si devices. Different resistive switching behaviors were observed for the different dopant concentrations in silicon BE. The device with the lower dopant impurity concentration had a more nonlinear response and a low operating current in the LRS, while also displaying self-compliance set switching; this is in comparison with the device with the higher impurity concentration. All these results reveal that the BE doping-controlled SiN*_x_* RRAM device in the MIS system would be suitable for high-density RRAM owing to its CMOS compatible structure and rectification and self-limiting properties.
